# The Mouse Papillomavirus Infection Model

**DOI:** 10.3390/v9090246

**Published:** 2017-08-30

**Authors:** Jiafen Hu, Nancy M. Cladel, Lynn R. Budgeon, Karla K. Balogh, Neil D. Christensen

**Affiliations:** 1 The Jake Gittlen Laboratories for Cancer Research, Hershey, PA 17033, USA; ncladel@gmail.com (N.M.C.); lrb11@psu.edu (L.R.B.); kkb2@psu.edu (K.K.B.); 2 Department of Pathology, Pennsylvania State University College of Medicine, Hershey, PA 17033, USA; 3 Department of Microbiology and Immunology, Pennsylvania State University College of Medicine, Hershey, PA 17033, USA

**Keywords:** the mouse papillomavirus, tissue tropism, anogenital, oral infection, skin carcinoma, pathogenesis, innate immunity, adaptive immunity, RNA sequencing, host defense

## Abstract

The mouse papillomavirus (MmuPV1) was first reported in 2011 and has since become a powerful research tool. Through collective efforts from different groups, significant progress has been made in the understanding of molecular, virological, and immunological mechanisms of MmuPV1 infections in both immunocompromised and immunocompetent hosts. This mouse papillomavirus provides, for the first time, the opportunity to study papillomavirus infections in the context of a small common laboratory animal for which abundant reagents are available and for which many strains exist. The model is a major step forward in the study of papillomavirus disease and pathology. In this review, we summarize studies using MmuPV1 over the past six years and share our perspectives on the value of this unique model system. Specifically, we discuss viral pathogenesis in cutaneous and mucosal tissues as well as in different mouse strains, immune responses to the virus, and local host-restricted factors that may be involved in MmuPV1 infections and associated disease progression.

## 1. Introduction

The papillomavirus research community has been searching for a mouse papillomavirus model since the identification of the cottontail rabbit papillomavirus in 1933 [1]. Over the years, a number of rodent papillomaviruses have been isolated, cloned and sequenced but none of them infected laboratory mouse strains [2]. In 1989, Tilbrook et al., identified papillomavirus DNA in hairless mouse tumors resulting from ultraviolet irradiation exposure [3]. They subsequently inoculated this mouse with the cell-free extract of these skin tumors and observed increased tumor incidence and degree of malignancy upon irradiation [4]. They demonstrated that the viral DNA shared homology with *Mastomys natalensis* papillomavirus DNA as well as with HPV11, -13, -16 and -18 by hybridization. The viral genome, however, was not isolated or sequenced [3,4]. In 2011, a mouse papillomavirus (subsequently labeled MmuPV1) was identified in a colony of nude (NMRI-Foxn1^nu^/Foxn1^nu^) mice in India [5]. The DNA sequence was reported in a subsequent publication [6]. A variant of MmuPV1 was later identified in a house mouse [7]. In the few years since it was first reported, MmuPV1 has become a valuable animal papillomavirus because it provides, for the first time, the opportunity to study papillomavirus infections in the context of a small common laboratory animal for which abundant reagents are available and for which many strains exist. Several groups, including our own, have established this mouse model system and have made significant progress in understanding molecular, virological, and immunological mechanisms of MmuPV1 infections in both immunocompromised and immunocompetent hosts [5,6,7,8,9,10,11,12,13,14,15,16,17,18,19,20,21,22,23]. In this review, we summarize studies using this mouse papillomavirus model over the past six years and share our perspectives on the value of this unique model system. Specifically, we discuss viral pathogenesis in cutaneous and mucosal tissues as well as in different mouse strains, immune responses to the virus, and local host-restricted factors that may be involved in MmuPV1 infections and associated disease progression.

## 2. Mouse Papillomavirus Exhibits both Cutaneous and Mucosal Tropism

**Figure 1:**
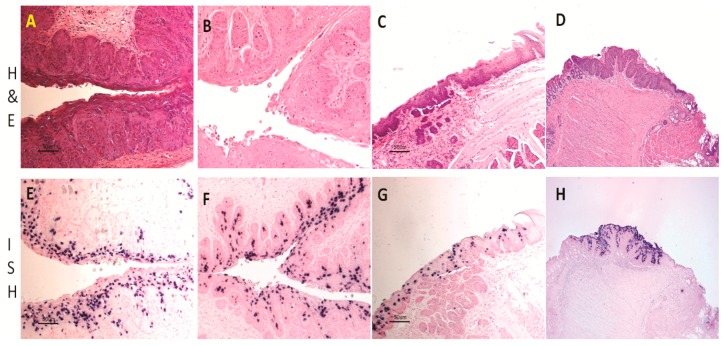
Histology of infected mucosal sites in Hsd:NU Foxn1^nu^ mice: the lower genital tract (**A**,**B**, 20×); the tongue (**C**, 20×); and the anal tract (**D**, 10×). Viral DNA was detected at the corresponding sites by in situ hybridization. Vaginal tract (**E**, 20×) and cervix (**F**, 20×) were positive for viral DNA. The Circumvallate papilla was the primary target for tongue infection (**G**, 20×). The transition zone of the anal tract was the most susceptible site for viral infection (**H**, 10×).

First reports of MmuPV1 identified the virus as strictly cutaneous [5]. Indeed, the lesions first observed were florid muzzle tumors [5]. Most laboratories have focused their work on cutaneous sites including the tail, the muzzle, the back, and the ear [11,13,14,15,16,17,18]. When compared with muzzle and tail sites, the back skin was the least susceptible site for the primary infections ([14]. Our recent work has supported this observation and has shown significantly less encapsidated DNA in back skin vis a vis in muzzle and tail sites (manuscript in press). Work in our laboratory showed, however, that the vaginal, anal and oral mucosae of immunocompromised Hsd:NU Foxn1^nu^, NU/J-Foxn1^nu^, and B6.Cg-Foxn1^nu^ mice are also easily infected [20,21,22,23]. Figure 1 is representative of mucosal lesions in Hsd:NU (Foxn1^nu^) nude mice.

Vaginal infections were later confirmed by other groups [8,11]. We are able to readily establish infections in these disparate mucosal tissues and follow the progress of disease by QPCR examination of viral DNA in lavage samples [21]. Cytology was also very useful to monitor infections in the lower genital tract [21,22]. These noninvasive methods have proved to be powerful tools in our laboratory as they allow not only for disease progression to be tracked longitudinally but also for maximal data to be obtained from a small number of animals. Histological analyses were also conducted following sacrifice of the animals. Using these tools, we have demonstrated that:

(1) The single circumvallate papilla at the back of the mouse tongue is preferentially targeted by the virus [20]. This observation provides opportunities to study viral infections at the back of the tongue, the site of an increasing number of papillomavirus-associated human tumors [24].

(2) The vaginal and anal tracts are highly susceptible to virus infections [21,22]. This observation provides a novel in vivo model to study both anal and genital infections concurrently in the same host. These studies may reveal information that could lead to better understanding and control of corresponding human infections and diseases.

(3) Primary infections at cutaneous sites can lead to secondary infections at mucosal sites [23]. In a similar manner, secondary cutaneous infections often follow primary infections in immunocompromised mice (manuscript in press). Thus, primary infection in the oral cavity or vagina can lead to secondary infections at skin sites on the back, muzzle or tail.

## 3. Certain Strains of Immunocompetent Mice Are Susceptible to MmuPV1 Infection

MmuPV1 was identified in a colony of NMRI-Foxn1^nu^/Foxn1^nu^ nude mice in India [5]. These mice were immunocompromised and there was interest in determining whether immunocompetent strains could also maintain infections. In this early report, the authors noted the development of small cutaneous papillomas on the back skin of immunocompetent S/RV/Cri-ba/ba (bare) mice [11,17]. These lesions regressed by eight weeks and no further analyses were conducted. Subsequently, Jiang et al. tested SKH1 (Crl: SKH1-Hrhr) mice and found that tail lesions in a subset of these animals persisted over time [18]. Uberoi et al. subjected FVB/NJ immunocompetent mice to UVB radiation and noted that, following treatment, ear lesions persisted although the mice were resistant to infection prior to irradiation [13]. In work submitted for publication from our laboratory, we observed that several immunocompetent mouse strains (e.g., C57BL6 and hairless SKH-1) mounted transient mucosal infections, which quickly regressed. However, the immunocompetent heterozygotes of inbred NU/J mice (Foxn1^nu/+^), outbred Hsd (Foxn1^nu/+^), and C57BL/6 (Foxn1^nu/+^) mice maintained persistent vaginal infections. Thus, it is clear that MmuPV1 infections are not restricted to immunocompromised mouse strains.

## 4. MmuPV1 Has Malignant Potential

### 4.1. Mouse Papillomavirus Oncogenes E6 and E7 Are Tumorigenic

**Figure 2:**
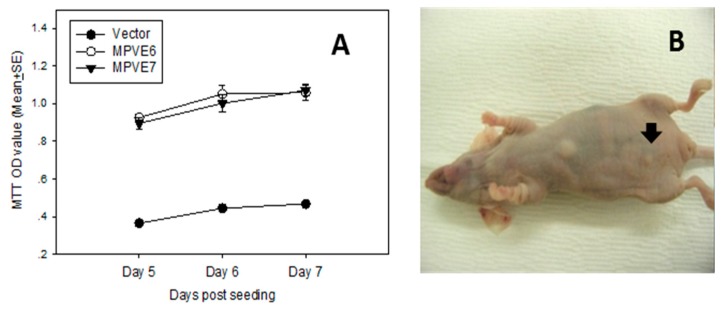

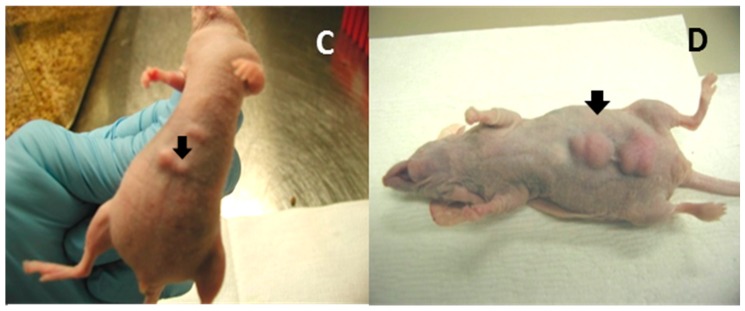
MmuPV1 E6 and E7 cloned into the expression vector (PCR3) were transfected into NIH3T3 cells under the selection of G418. The stably transfected cells were tested for proliferation in vitro. Both E6 and E7 showed significantly higher proliferative activity (**A**); E6 and E7 stably transfected NIH3T3 cells also showed tumorigenicity in vivo ((**C**,**D**), respectively, see arrows); and the vector control showed minimal disease (**B**).

As in many other papillomaviruses, the mouse papillomavirus also contains two putative viral oncogenes, E6 and E7 [6]. We have tested the tumorigenicity of these proteins in vitro and in vivo using standard methods that we have previously described [25]. Both gene products showed significantly higher proliferative activity in vitro (Figure 2A) and tumorigenicity in vivo (Figure 2C,D). A recent study showed that MmuPV1E6 shared some biochemical and functional characteristics with cutaneous HPV8 E6 including inhibiting NOTCH and TGF-β signaling as well as contributing to delayed differentiation and prolonged survival of differentiated keratinocytes [16]. MmuPV1 E6 mutants also failed to induce tumor growth in nude mice indicating that E6 contributes to papillomavirus pathogenesis and carcinogenesis [16]. White et al. noted that MmuPV1 E7 bound PTPN14, a classical nontransmembrane protein tyrosine phosphatase (PTP), as did numerous oncogenic HPV E7s [26]. These observations suggest that E7 may share oncogenic properties with the high-risk human papillomavirus E7. Much work remains to be done to further clarify the roles of MmuPV1 E6 and E7.

### 4.2. Cutaneous Lesions Can Develop into Cancers

Sundberg et al. reported the development of locally invasive poorly differentiated carcinomas on the dorsal skin of B6.Cg-Foxn1^nu^/Foxn1^nu^ mice [11]. They noted that the tumors resembled trichoblastomas seen in humans [27]. Uberoi et al. subjected FVB/NJ immunocompetent mice to UVB radiation and subsequently infected the mice at ear and tail sites [13]. Many of the mice developed ear lesions, some of which progressed to squamous cell carcinomas. This was the first report of an MmuPV1 cancer in an immunocompetent mouse. In our laboratory, we have detected two different and spontaneously developing cutaneous skin carcinomas as secondary skin infections in Hsd:NU Foxn1^nu^ nude mice. Taken together, these findings clearly demonstrate the malignant potential of MmuPV1 at cutaneous sites.

### 4.3. Mucosal Lesions Can Develop into Cancers

In data under review for publication, our laboratory has shown that both homozygous NU/J mice (immunocompromised) and their immunocompetent heterozygous counterparts develop carcinoma in situ in MmuPV1-infected vaginal tissues. These carcinomas were detected at 7.5 months post infection. These findings and those above suggest that MmuPV1 may prove to be a useful model to study papillomavirus-associated malignant progression at both cutaneous and mucosal sites in a tractable animal model.

## 5. Immune Responses to MmuPV1 Infections

Both adaptive and innate immunity play a role in MmuPV1 infections at both cutaneous and mucosal sites. Studies of adaptive immune responses have been focused on T-cell mediated immune responses in cutaneous infections [13,15,17,18]. Initial innate immune responses have been studied in both cutaneous and mucosal infections in our laboratory.

### 5.1. T- and B-Cell Mediated Immunity in the Control of MmuPV1 Infections

**Figure 3:**
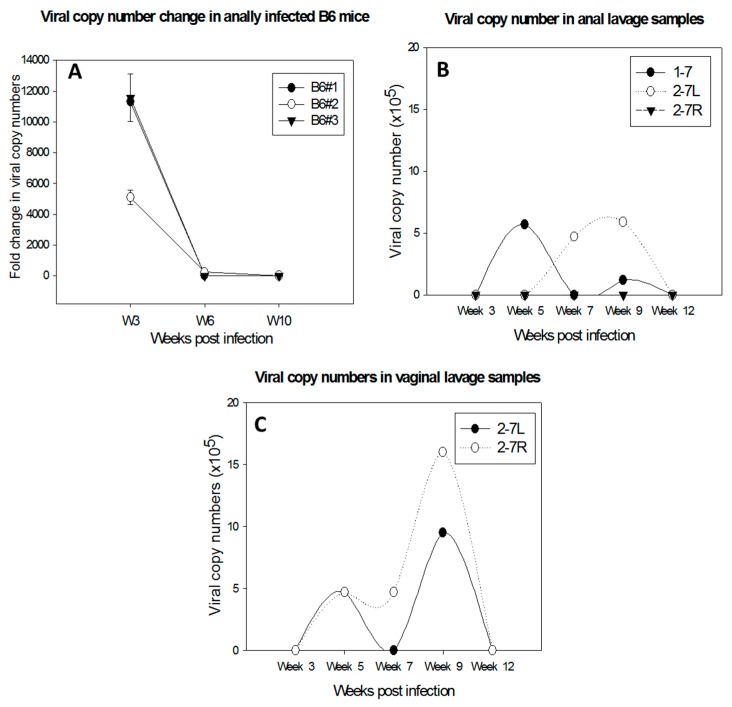
Delayed regression of anal infections was observed in C57BL/6 mice depleted of both CD4 and CD8 T cells (**A**). Viral DNA was detected in lavage samples of the anal tract of one male (1–7) and two females (2–7L and 2–7R) (**B**); and the lower genital tract of 2–7L and 2–7R of Ifnar^−/−^ mice (**C**). In contrast, viral infections were cleared in the wild type B6 mice before Week 5 post-infection.

Several immunocompetent mouse strains including C56BL/6, FVB/NJ, and SKH-1 have been tested for susceptibility to MmuPV1 infections at cutaneous sites [8,11,15,17]. Both CD4 and CD8 T cells have been found to play a crucial role in the control of papillomavirus infection at these sites, although neither CD4 nor CD8-knockout or -depletion led to visible disease in these immunocompetent mice [15,17,18]. Further studies demonstrated that a strong E6- and E7-specific CD8+ T cell response is correlated with viral clearance and tumor regression in vaccinated mice [18]. Specifically, transferred E6/90-99 specific CD8 T cells can prevent the development of tumor growth in MmuPV1-infected athymic mice [17]. We have observed persistence and delayed regression of anal infections in C57BL/6 mice depleted of both CD4 and CD8 T cells (Figure 3A). Passive immunization with serum from virus-particle immunized mice provided strong protection against primary viral infection [8]. Our recent studies also demonstrated complete protection at both cutaneous and mucosal sites as a result of passive immunization with a neutralizing monoclonal antibody in athymic mice (unpublished observations). These findings suggest that both T- and B-cell mediated immune responses play a critical role in the clinical outcome of MmuPV1 infections.

### 5.2. Innate Immunity in the Control of MmuPV1 Infection at Mucosal Sites

Type I interferon pathways are the first defense against pathogen invasion [28,29,30]. A previous study failed to detect visible disease at cutaneous sites in ifnar^−/−^ mice [17]. In contrast, we found a prolonged time to regression at the anogenital (vaginal and anal) sites in these mice compared to wild-type mice (Figure 3B,C). The infections were detectable up to three months post infection in contrast to infections in wild type animals in which the viral DNA usually became undetectable by week four post infection (our unpublished observations).

#### 5.2.1. Neutrophils and NK Cells Are Associated with Decreased Local MmuPV1 Mucosal Infection in Immunocompetent Heterozygous (Foxn1^nu/+^) NU/J Mice

Innate immune cells including NK cells and neutrophils are important in host defense against the early stages of viral infections. Previous studies demonstrated that adaptive immunity is sufficient to eliminate skin MmuPV1 infections in immunocompetent mice [15,17]. We examined both neutrophils and NK cells in situ in infected mucosal and cutaneous tissues of both immunocompetent heterozygous NU/J mice (Foxn1^nu/+^) and immunocompromised homozygous (Foxn1^nu/nu^) NU/J mice. We found that increased numbers of NK cells were observed in the tail tissues of the heterozygous NU/J mice (with minimal infections) relative to those detected in the homozygotes with visible lesions (data not shown). Tissue resident NK cells are reported to be very different from conventional splenic NK cells in that they produce cytokines TNFα and GM-CSF rather than of IFNγ [31]. Although cytokines are key players in numerous inflammatory processes and the production of cytokines is under tight genetic control [32], this difference in resident NK cells may result in differential local cytokine production leading to tissue-specific disease outcome.

Immunocompetent (Foxn1^nu/+^) and immunocompromised (Foxn1^nu/nu^) NU/J mice showed persistent infections in vaginal tissues. We detected higher numbers of infiltrating neutrophils in the vaginal tissues of the immunocompetent (Foxn1^nu/+^) NU/J mice than in those of immunocompromised (Foxn1^nu/nu^) mice. The homozygous (Foxn1^nu/nu^) mice had more severe disease indicating that neutrophils may have contributed to the control of disease in the immunocompetent (Foxn1^nu/+^) mice (manuscript in preparation). Collectively, our findings suggest that neutrophils and NK cells may be associated with reduced MmuPV1 infection in immunocompetent mice. The role of these and other immune cells in MmuPV1 infection and persistence needs further investigation.

#### 5.2.2. RNA Sequencing Data Support the Involvement of NK Cells and Neutrophils as Well as Type I IFNs in MmuPV1-Infected Tissues

Recently, we conducted genome-wide RNA sequencing on different tissues with or without MmuPV1 infection. In agreement with findings in HPV-associated human studies, many molecules in different signaling pathways associated with antiviral activities, cell growth and differentiation, cancer development, and inflammation were dysregulated in the infected tissues [33,34,35,36,37]. Host defense factors including β-defensins [36,38,39,40,41] and TLR5 [42,43] were associated with MmuPV1 infection (Table 1). Type I interferon (IFNs) are the most potent known antiviral factors limiting the replication and spread of most viruses [30,44,45]. Significant changes were found in type I IFNs as well as IL-12, IL-15, and IL-18 (Table 1). These molecules are produced by macrophages and dendritic cells and are critical for NK-cell maturation and regulation of NK-cell function [46,47,48,49]. CXCR2 expressed by circulating neutrophils was upregulated in infected muzzle tissues (Table 1). We have also detected significant changes of several interferon-stimulated genes (ISGs). For example, Stat3 and Stat6, which promote innate antiviral responses and contribute to the detrimental effects of viral infection, were upregulated in MmuPV1-infected muzzle and tongue tissues (Table 1). Other ISGs including Trim proteins (Trim 23 and Trim 29) were dysregulated in infected tissues (Table 1). We also detected downregulation of CD53, a pan-leukocyte surface glycoprotein proposed to play an important role in thymopoiesis and leukocyte signal transduction in all infected tissues [50].

## 6. Other Host-Restricted Factors in Local MmuPV1 Infections

Besides the involvement of innate and adaptive immunity in papillomavirus infections, other host-restricted factors may play a role in disease outcome. A previous study showed that NOD/SCID mice, a strain that is deficient in T-, B-, and NK cells, were resistant to cutaneous MmuPV1 infections [11]. In agreement with this finding, we detected a single small tail lesion on one of seven infected NOD/SCID mice although all infected sites were positive for viral DNA (manuscript under review). This finding suggests that a latent or subclinical infection may have been established in these infected tail tissues and that additional host defenses may have played a role in the control of cutaneous infections in this strain.

We and others have observed site specific infections in other mouse strains. For example, the inbred NU/J nude mice, which manifested minimal cutaneous disease relative to that in outbred Hsd:NU mice, showed advanced mucosal infections. We and others observed that the back skin is less susceptible to viral infection when compared with the muzzle and tail in the same animal [14]. In addition, we have shown that viral encapsidation is significantly reduced in back skin relative to other cutaneous sites (manuscript in press). To understand the site-specificity, Sundberg et al. conducted transcriptome assays in different skin papillomas [11]. While they found dysregulation of several skin cancer-associated genes in papilloma tissues, they also found a significant difference in gene expression in different skin sites supporting the concept that the local environment may contribute to the disease outcome at these sites [11]. In another study, the authors noted that the tail showed less disease than the ear skin in UV-irradiated mice [13]. All these findings suggest local host defense factors may have contributed to disease outcome in these different mouse strains. More thorough molecular and genetic studies should provide new evidence of the site-specific host control in viral infections.

## 7. Conclusions and Future Directions

The discovery of MmuPV1 is very recent. In the short time since it was first reported, several groups have made significant findings with respect to the immunology, molecular biology, and tropism of the disease [5,6,7,8,9,10,11,12,13,14,15,16,17,18,19,20,21,22,23]. The findings have set the stage for some very useful new models to investigate papillomavirus disease in vivo. They also introduce new opportunities to better refine our understanding of tissue-specific immune responses to papillomavirus infections. Among the most important findings and their implications are the following.

(1) The first small animal model to study papillomavirus infections and associated diseases in anogenital and oral tissues:

More than 66% of cervical cancers are associated with PV. Cervical cancers create an enormous medical burden for the world’s women. More than 250,000 individuals die each year of the disease [51,52]. Current prophylactic vaccines have no effect on existing disease and so a therapeutic vaccine would represent an important advancement [53]. With the finding that certain immunocompetent strains of mice support persistent vaginal MmuPV1 infections, the model is well-placed to test potential therapeutic vaccines [54].

PV-related oral cancers are on the rise in younger men [55,56]. They present a major challenge to the medical community and result in considerable morbidity for the patients. A suitable preclinical model of oral PV-associated disease is therefore in great demand. The finding that the circumvallate papilla of the mouse tongue is a preferred site for infection suggests that the MmuPV1/mouse model could be ideal for the study of papillomavirus-related oral cancers in humans, in which cancers tend to occur at the back of the tongue and in the tonsillar region [57].

Anal cancers are on the rise in women [58]. The reasons are poorly understood and few interventions are available. MmuPV1 infects the anal canal, especially the transition zone. The MmuPV-1 mouse model thus could provide a much-needed model to study these enigmatic infections.

(2) A small animal model to study host defense against papillomavirus infections:

T- and B-cells have been shown to be critical factors in generating immunity to MmuPV1. However, the fact that not all mouse strains deficient in T- and B-cells develop MmuPV1 lesions supports the idea that other immune components and pathways can play a role. Our early work has shown possible roles for neutrophils and NK cells and both RNAseq data generated in our laboratory and the microarray data of Sundberg et al. support this hypothesis as well [11]. We expect that a more complete analysis of the RNA sequencing and array data will suggest other avenues for investigation and validation. The availability of many genetically-modified mice will also allow for the expanded investigation of host factors in viral infections.

(3) A small animal model to study papillomavirus-associated tissue tropism:

Tissue tropism has always been of interest to researchers in the papillomavirus field. Most papillomaviruses display either cutaneous or mucosal tropism and even within those broad categories only specific tissues are commonly targeted [51]. MmuPV1 is different in that it displays both cutaneous and mucosal tropism [11,23]. The tissues of different strains can be differentially susceptible to the virus infections and this provides a tool to plumb local immune and other factors contributing to tissue tropism. We anticipate that high throughput analyses such as RNAseq will be instrumental in helping to elucidate the molecular and cellular components of PV tissue tropism.

(4) A small animal model to study papillomavirus-associated skin cancers:

Cutaneous cancers are sometimes associated with papillomaviruses [59]. MmuPV1 lesions have been shown to undergo malignant transformation in immunocompromised animals [11] as well as in immunocompetent irradiated mice [13] and so the MmuPV1 model may become a useful tool to study cutaneous skin cancer disease and progression.

(5) A small animal model to study papillomavirus-associated transmission:

Our recent studies demonstrated that genital tissues of both males and females are susceptible to viral infections. In addition, our studies have shown that the development of secondary lesions resulted from virus shedding from primary infections [21,23]. These observations suggest that the MmuPV1 model will be useful to study both horizontal (between partners) and vertical (mother to child) transmission of virus. HPV-induced Recurrent Respiratory Papillomatosis (RRP) is believed to result from HPV transmission from mothers to newborns as a result of passage through the birth canal [60]. RRP is a devastating pathological condition in children. It is characterized by the recurrent appearance of wart-like lesions in the respiratory tract, particularly at the larynx and vocal cords [61]. These patients must undergo repeated surgery or other invasive treatment to manage the disease. There is a great need for model systems to study papillomavirus-associated vertical transmission.

**Figure 4:**
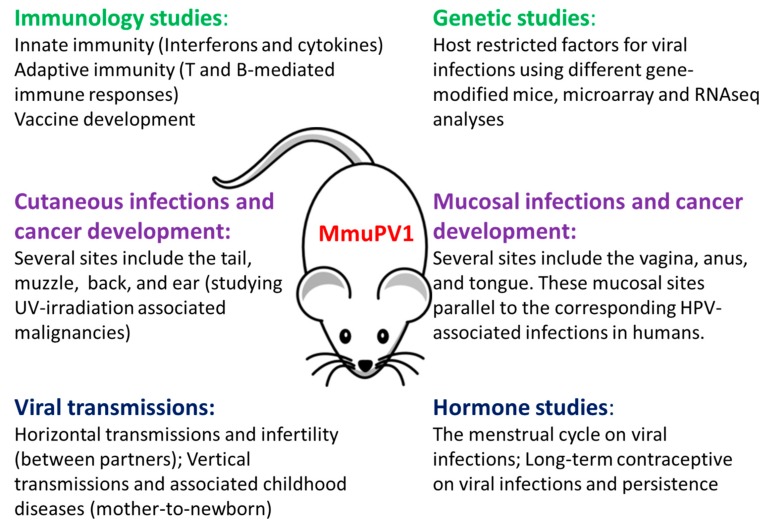
The potential application of the MmuPV1/mouse model.

(6) A small animal model to study the role of the menstrual cycle and contraceptives in papillomavirus-associated diseases:

The influence of the menstrual cycle and contraception on other viruses such as genital herpes and zika has been reported [62,63,64,65]. Whether the menstrual cycle and contraception play a role in genital papillomavirus infections is of great practical interest. We demonstrated in our previous studies that highest viral titers was detected at the estrus stage [21]. Whether contraception plays a role in viral susceptibility and persistence is under investigation in the laboratory. The MmuPV1 vaginal model will be an excellent tool to study the interplay between viral infection, the menstrual cycle, and contraceptive use. Figure 4 illustrates in graphic form the potential of the MmuPV1 model for multiple studies in situ. The model is a major step forward in the study of papillomavirus disease and pathology.

## Figures and Tables

**Table 1:** Some significantly changed molecules in MmuPV1-infected tissues.

Transcripts	MmuPV1 Infected Tissues		
	Muzzle	Tongue	Vagina
IL15	Down	Down	N.S.
Il1rn	UP	UP	N.S.
Il4ra	N.S.	UP	UP
IFNar1	N.S.	UP	N.S.
Ifi27l2b	N.S.	Down	Down
Ifi27	Down	N.S.	N.S.
Ifit2	N.S.	N.S.	Down
TLR5	N.S.	UP	N.S.
CXCR2	UP	N.S.	N.S.
CD53	Down	Down	Down
Stat3	UP	UP	N.S.
Stat6	UP	UP	N.S.
Trim23	Down	Down	N.S.
Trim29	UP	N.S.	UP
Defb4	Down	Down	N.S.
Defb6	Down	Down	N.S.

N.S. Not significant between the infected vs. non-infected tissues.
